# VaxArray potency assay for rapid assessment of “pandemic” influenza vaccines

**DOI:** 10.1038/s41541-018-0080-6

**Published:** 2018-10-08

**Authors:** Rose T. Byrne-Nash, David F. Miller, Katie M. Bueter, Jacob H. Gillis, Laura R. Kuck, Kathy L. Rowlen

**Affiliations:** InDevR Inc., 2100 Central Ave., Suite 106, Boulder, CO 80301 USA

## Abstract

The VaxArray Influenza Pandemic HA (VXI-pHA) potency assay is a multiplexed sandwich immunoassay that consists of nine broadly reactive yet subtype-specific monoclonal capture antibodies printed in microarray format and a suite of fluor-labeled secondary antibodies that were selected to probe conserved HA epitopes. VXI-pHA was designed to optimize the probability that the ready-to-use assay would work for the most concerning, emergent influenza A strains, eliminating the need for the time-consuming process of reference reagents production. The performance of this new potency test was evaluated using a panel of 48 potentially pandemic strains of influenza viruses and vaccines spanning 16 years of antigenic drift, including the most recent pre-pandemic vaccine being developed against the “5^th^ wave” A/H7N9 virus. The VXI-pHA assay demonstrated coverage of 93%, 92%, and 100% for H5, H7, and H9 antigens, respectively. The assay demonstrated high sensitivity with linear dynamic ranges of more than 150-fold and quantification limits ranging from 1 to 5 ng/mL. For three production lots of H7N9 monobulk drug substance, the assay exhibited excellent accuracy (100 ± 6%) and analytical precision (CV 6 ± 2%). The high assay sensitivity enabled robust detection and quantification of hemagglutinin in crude in-process samples and low-dose, adjuvanted vaccines with an accuracy of 100 ± 10%.

## Vaccines potency: A new specific assay for potential pandemic influenza

Potency of inactivated influenza vaccines is determined based on the viral hemagglutinin (HA) content in the vaccine preparation. Specific assays need to be rapidly available to avoid a delay for pandemic vaccine production. In this study, led by Kathy Rowlen, researchers from InDevR in Colorado developed an immunoassay for potency testing of pandemic influenza vaccines. The VaxArray assay utilizes a panel of monoclonal antibodies and recognizes 94% of the tested H5, H7, and H9 antigens representing 16 years of circulating influenza viruses. VaxArray shows high specificity for the tested HAs and recognizes only correctly folded HA antigen, which is crucial to accurately determine vaccine potency. Furthermore, the assay displays high sensitivity and accuracy, even for crude in-process samples containing contaminants from production or for low dose, adjuvanted samples. The combination of broad reactivity and high specificity for potentially pandemic HAs makes VaxArray a promising tool for pandemic preparedness.

## Introduction

To help prevent or mitigate the severity of a potentially devastating influenza pandemic, public health agencies and vaccine manufacturers must be able to rapidly define, develop, manufacture, and release “pandemic” vaccines. In the spring of 2009, the emergence of the pandemic H1N1 virus revealed bottlenecks in the process. One of those bottlenecks originated with the potency assay used to release influenza vaccines, single radial immunodiffusion (SRID). Specifically, the hemagglutinin protein (HA) from the H1N1 pdm2009 strain was particularly sensitive to bromelain digestion, which delayed the production of antiserum that serves as one of the reference reagents in SRID.^1^ This critical limitation in the creation and qualification of reference reagents for the SRID potency assay contributed to a delay in the release of an appropriate H1N1 pdm2009 vaccine. As a result, the World Health Organization (WHO) and other agencies have encouraged the development of alternative methods for more efficient potency testing of pandemic vaccines.^1–4^

There is also a drive for improved sensitivity in potency testing for pandemic vaccines. While most seasonal influenza vaccines are released with ~30 µg/mL HA, during a pandemic influenza vaccines are likely to be adjuvanted to allow for dose-sparing formulations. Low-dose influenza vaccines can contain 5 µg/mL HA or less, which is at or below the limit of detection for SRID. Furthermore, SRID is inherently incompatible with adjuvants that contain squalene or aluminum hydroxide.^5^

To address these shortcomings of SRID, we developed the VaxArray Influenza Pandemic HA Potency Assay (VXI-pHA). The intent of the VXI-pHA test is to make a potency assay available—in advance of a pandemic—for influenza viruses that are considered by the WHO to most likely result in a pandemic. The VaxArray potency testing platform is based on a multiplexed immunoassay.^6,7^ The antibodies used in the VXI-pHA assay presented here were generated by scientists at the Center for Biologics Evaluation and Research (CBER) within the US Food and Drug Administration, as part of their contribution to the Health and Human Services’ interagency Influenza Vaccine Manufacturing Improvement Initiative. The monoclonal antibodies (mAb) were selected for broad coverage of potentially pandemic subtypes: specifically, H5, H7, and H9 subtypes. While it is not possible to guarantee that the assay would be responsive to a new H5, H7, or H9 influenza virus, the broad reactivity of the mAbs included in this panel represents a robust attempt to prepare in advance. As demonstrated here, the VXI-pHA test is 24× faster than SRID (2 vs 48 h), reagent sparing, compatible with common adjuvants, and does not require the generation of antisera.

## Results

**Fig. 1:**
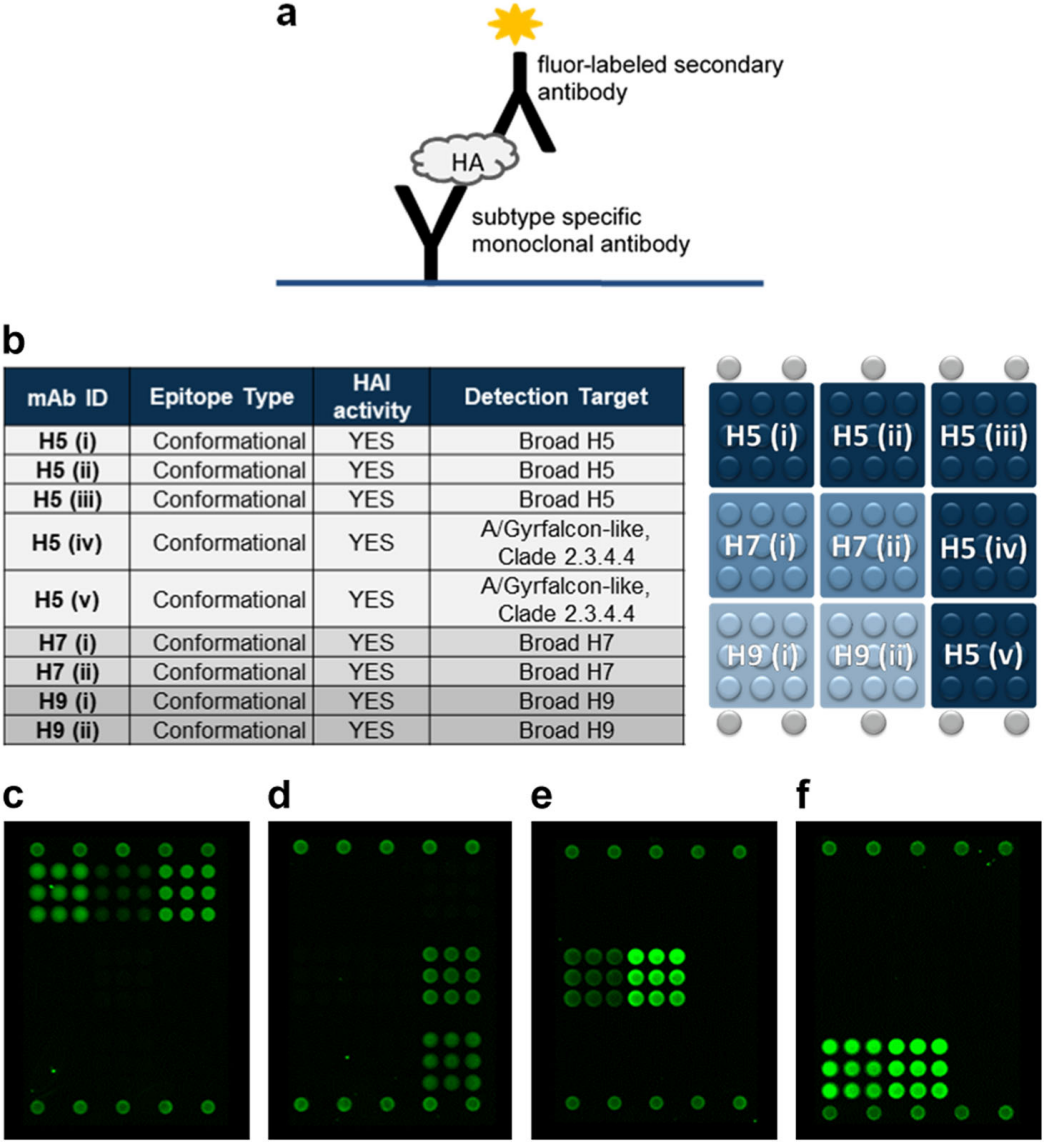
VaxArray influenza pandemic HA potency assay (VXI-pHA). **a** Illustration of the immunoassay principle. **b** Schematic of the VXI-pHA array layout of subtype-specific antibodies to A/H5, A/H7, and A/H9 subtypes. The array contains nine replicate spots (~200 µm in diameter) of each monoclonal antibody. Table provides epitope information and reactivity for each monoclonal antibody. Representative fluorescence images for the following vaccines on the VXI microarray: **c** A/Vietnam/1203/2004 from H5 Clade 1 (egg derived, CBER Reference Antigen, Lot #50), **d** A/Gryfalcon/Washington/41088-6/2014 from H5 Clade 2.3.4.4 (egg derived, IRR, Cat # FR-1447), **e** A/Guangdong/17SF003/2016 from the H7N9 subtype (rHA), and **f** A/chicken/Hong Kong/G9/1997 from H9 Clade G1 (NIBSC, Lot # 08/228)

The VXI-pHA assay was previously developed to provide broad coverage and highly sensitive detection of potentially pandemic influenza A H5, H7, and H9 subtype virus HA. The resulting assay, VXI-pHA, is a multiplexed sandwich immunoassay that consists of subtype-specific monoclonal antibodies on a microarray platform that capture HA protein before a fluor-labeled secondary antibody is added for detection (Fig. 1a). The subtype-specific monoclonal antibodies are printed in array format (Fig. 1b) in replicates of 9 on a 16-well glass slide. In work done prior to the studies outlined in this manuscript, a large panel of monoclonal antibodies licensed from the CBER was screened for coverage and sensitivity (data not shown). A minimum of two different mAbs was selected for each subtype to optimize coverage within a given subtype (Fig. 1b). The H5(i–iii) antibodies, with broad detection of pre-2014 H5 antigens (Fig. 1c), do not detect the recently emerged H5N8 A/Gryfalcon-like strains (Fig. 1d) from Clade 2.3.4.4. For this reason, we included the H5(iv) and H5(v) antibodies, which were raised against A/Gryfalcon-like HA antigens.

### VXI-pHA has high coverage and specificity for H5, H7, and H9 antigens produced across multiple production platforms

**Fig. 2:**
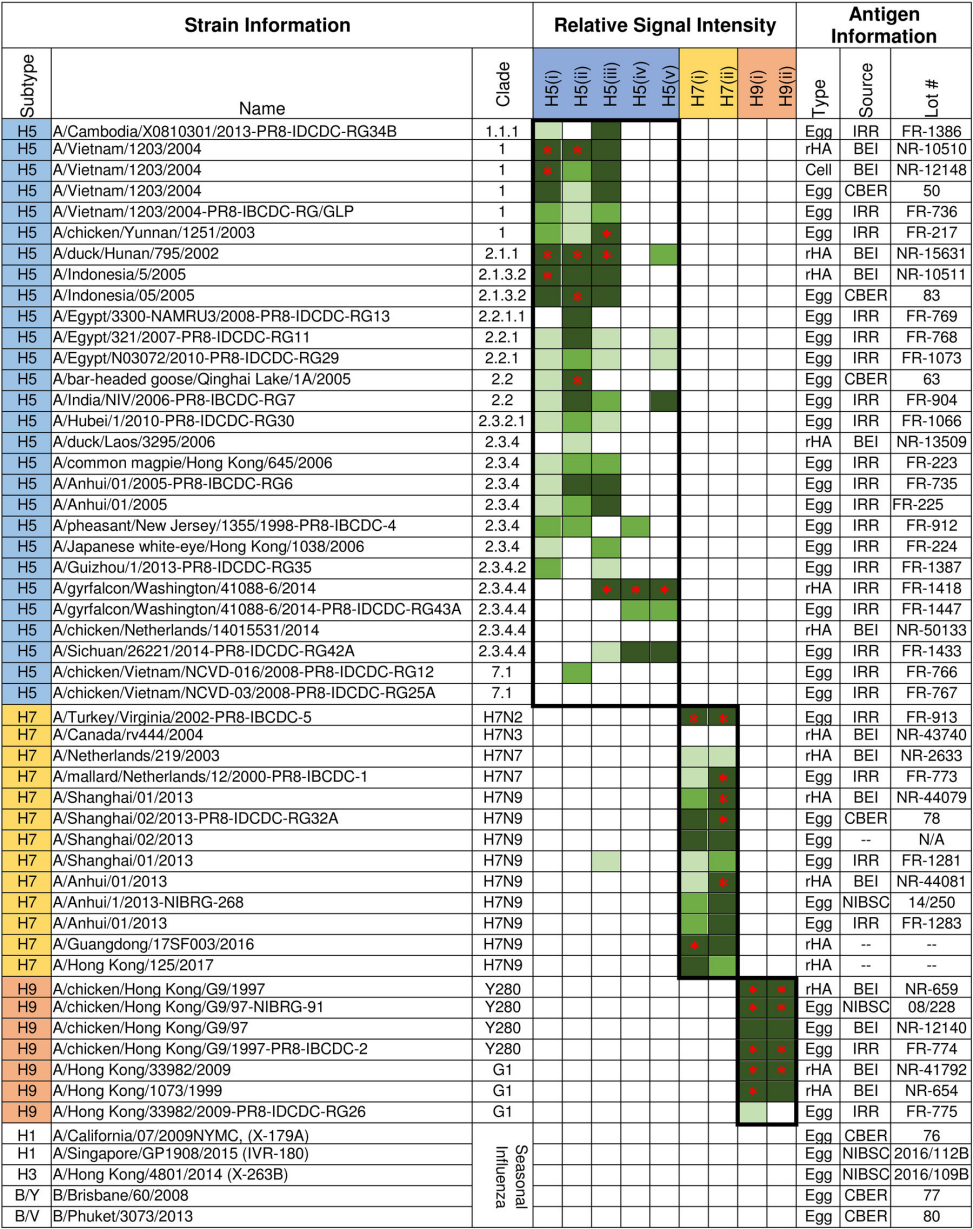
Relative signal intensities for VXI-pHA capture antibodies against a panel of pandemic antigens. The listed panel of antigens were tested on VXI-pHA. White/empty boxes indicate signal intensity below the 3× background intensity cutoff, light green indicates signal intensity between 3× and 10× background intensity, green indicates between 10× and 20× background, dark green indicates between 20× and 40× background, and dark green with red asterisk indicates 40× to fluorescence saturation

A panel of 48 antigens spanning the H5, H7, and H9 subtypes was tested in the VXI-pHA assay at relatively high concentrations (5 µg/mL for recombinant HA (rHA) and 0.5 µg/mL for all other antigens) to evaluate coverage within a subtype, specificity for a specific subtype, and relative sensitivity. These antigen concentrations were selected because previously reported studies found that rHA antigens are more compatible with the VaxArray platform at slightly higher concentrations than egg-derived antigens.^7^ The signal intensity for each capture antibody when exposed to each antigen was quantified and categorized as below the limit of detection (defined by 3× the background), 3−10× above background, 10×–20× above background, 20−40× above background, or above 40× background. The results of this study are reported in Fig. 2 and are further discussed in the paragraphs below. It is important to note that this analysis was performed as a qualitative assessment of the coverage and specificity of each capture antibody within the VXI-pHA assay. Because the antigens differed in age and storage conditions, we cannot compare signal intensities between samples and make quantitative conclusions with any confidence since each antigen likely has a varying level of degradation.

**Fig. 3:**
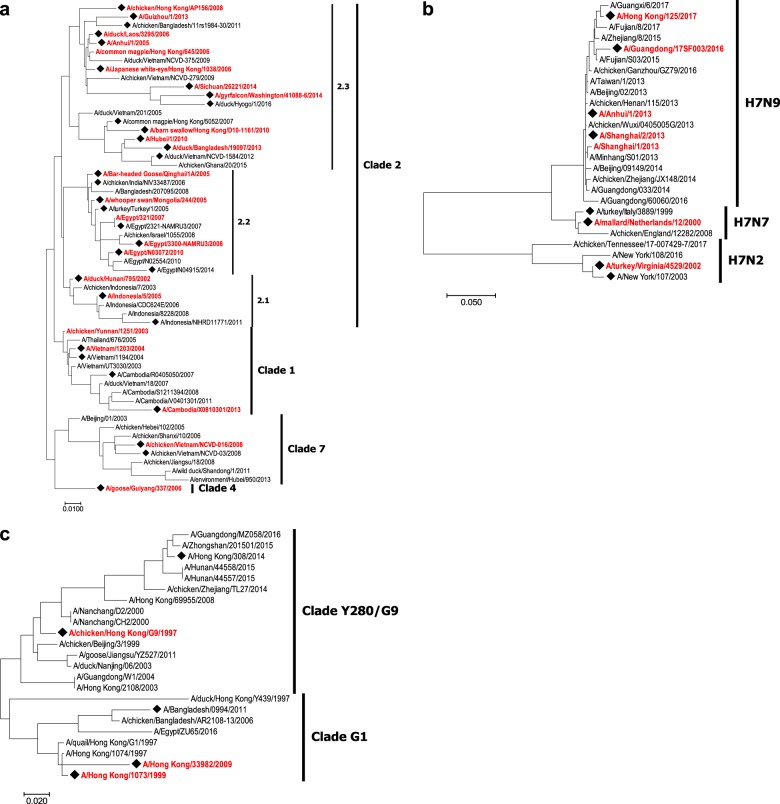
VXI-pHA coverage across the H5, H7, and H9 subtypes. Phylogenetic trees of potentially pandemic vaccine-relevant strains of A/H5 **a**, A/H7 **b**, and A/H9 **c** influenza. Candidate vaccine virus strains (CVVs) are distinguished by a ◆. Strains that have been tested on the VXI-pHA assay and have been positively identified are marked by red text. Strains in black have not yet been tested

The VXI-pHA assay detected 26/28 (93%) of the H5 antigens included in the panel with sufficient signal at assay relevant concentrations (Fig. 2). To assess the coverage of the VXI-pHA potency assay across the H5 subtype, a phylogenetic analysis was performed using the strain information for the 26 detected H5 antigens (Fig. 3a). The assay coverage broadly spanned the A/H5 phylogenetic tree, with confirmed detection of clades 1, 2, 4, and 7, which are the focus of candidate vaccine virus (CVV) development.^8^ CVVs are strains identified by the WHO for use in future vaccine development. The detection of all tested CVVs suggests that VXI-pHA would be able to quickly respond to the call for a new pandemic vaccine, which would likely be developed to one of these CVV strains. Additionally, the antigens detected represent 16 years of evolutionary time, from 1998 to 2014, suggesting the capture antibodies are probing fairly conserved epitopes and would be less likely to be affected by antigenic changes in novel strains.

The two H5 strains not detected at signal intensity greater than 3× background were A/chicken/Vietnam/NCVD-03/2008-PR8-IDCDC-RG25A, an egg-based H5N1 antigen from the International Reagent Resource (IRR), and A/chicken/Netherlands/14015531/2014, a recombinant H5N8 antigen from the Biodefense and Emerging Infections Research Resources Repository (BEI). In product literature for the rH5 antigen, it is mentioned that the antigen is not active in hemagglutination assays, suggesting that this antigen is conformationally defective. It is possible that the A/chicken/Vietnam/NCVD-03/2008-PR8-IDCDC-RG25A antigen was also compromised given that the assay did detect a similar reassortant, A/chicken/Vietnam/NCVD-016/2008-PR8-IDCDC-RG12.

The H7 capture antibodies detected 12 of the 13 H7 antigens tested (92%) within the antigen panel (Fig. 2). The H7 capture antibodies detected antigens spanning the phylogenetic tree for the H7 subtype (Fig. 3b), including the most recently emerged H7N9 strains as well as more distant A/H7 viruses dating back to 2000. All four of the CVVs tested were detected by the assay, including the latest A/Guangdong and A/Hong Kong CVVs that were recently announced.^8^ The one sample not detected by the assay was an H7N3 A/Canada/rv444/2004 recombinant HA sample. This sample is discussed further below.

Of the seven H9 antigens tested, the VXI-pHA assay detected seven (100%) (Fig. 2) with strains spanning two clades (Fig. 3c) and 12 years. While there were few antigens available for evaluation, likely due to less emphasis being put on H9 strains as potential pandemic strains, the two VXI-pHA capture antibodies demonstrated good coverage.

To assess the compatibility of the VXI-pHA assay with vaccines produced using different production platforms, we screened antigens derived from egg-based, cell-based, and recombinant technologies. The VXI-pHA assay detected 86% (6/7), 83% (5/6), and 100% (3/3) of the non-egg-derived H5, H7, and H9 antigens, respectively. All five H5 capture antibodies were capable of detecting non-egg-derived samples. As an example of direct comparison, two A/Indonesia/05/2005 antigens, one recombinant from a baculovirus system and one egg-propagated, each demonstrate high signal intensities on antibodies H5(i), H5(ii), and H5(iii) (Fig. 2). The one non-egg-derived H7 antigen that was not detected was an A/Canada/rv444/2004 H7N3 recombinant antigen. Because there was only a single H7N3 antigen available for testing, it is not clear if the assay is not compatible with H7N3 antigens in general, recombinant H7N3 antigens, or if there was an issue with antigen stability.

The data presented in Fig. 2 were used to evaluate each capture mAb for specificity, which was defined as the number of subtype-specific detection events (signal over 3× background) divided by the total number of detection events for that antibody. For this study, seasonal HA antigens were included to determine whether or not the pandemic capture mAbs had any cross-reactivity with seasonal antigens. Four of the five H5 capture mAbs detected only H5 and did not detect H7, H9, H1, H3, or influenza B HA antigens tested, demonstrating 100% specificity to H5 antigen. The H5(iii) capture mAb detected one whole virus H7N9 A/Shanghai/1/2013 antigen (Fig. 2) at 3–10× background signal, resulting in a corresponding specificity of 95%. All other VXI-pHA capture mAbs, H7(i–ii) and H9(i–ii), demonstrated 100% specificity with no cross-reactivity with other pandemic or seasonal subtypes.

### The linear dynamic range of VXI-sHA is more than 150-fold

**Fig. 4:**
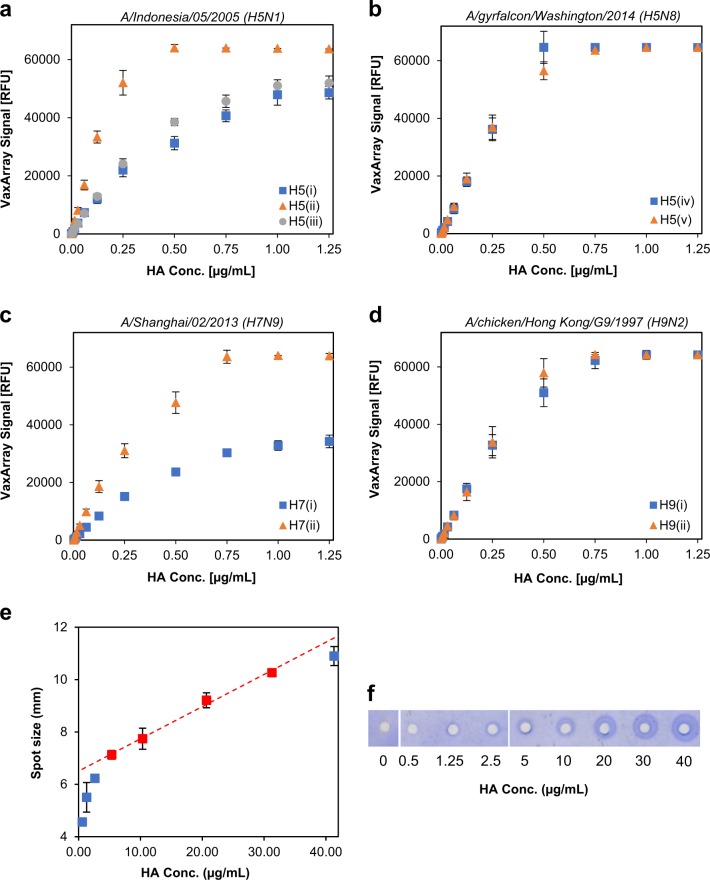
Linear dynamic range comparison of VXI-pHA and SRID. Serial dilutions of four antigens were analyzed by VXI-pHA. VXI-pHA response curves for A/H5N1 A/Indonesia/05/2005 (CBER, Lot #83) **a**, A/H5N8 A/gyrfalcon/Washington/41088-6/2014 (IRR, FR-1418) **b**, A/H7N9 A/Shanghai/02/2013 (CBER, Lot #78) **c**, and A/H9N2 A/chicken/Hong Kong/G9/1997 (NIBSC, Lot # 08/228) **d** antigens for each corresponding capture antibody are shown. Error bars represent the standard deviation of the 9 antibody spots for the corresponding capture antibody for each array. **e** Serial dilutions of H7N9 A/Shanghai/02/2013 (CBER, Lot #78) were performed and analyzed by SRID using appropriately matched antisera.The response curve for H7N9 A/Shanghai/02/2013 (CBER, Lot #78) is shown. Error bars represent the standard deviation of the 3 replicate measurements for each antigen concentration. Red points lie within the linear quantification range while blue points lie outside of the range and deviate significantly (*p* < 0.05) from the linear regression (red-dotted line). **f** One SRID replicate spot for each of the dilutions analyzed is shown for comparison

Thirteen-point serial dilutions of H5, H7, and H9 reference antigens with known HA concentrations were tested by the VXI-pHA assay over a range of 0.001–1.25 µg/mL to evaluate each capture mAb for linear dynamic range (LDR) as well as upper and lower limits of quantification. All of the VXI-pHA capture antibodies produced robust, linear curves in response to increasing antigen concentrations (Fig. 4a–d). The H5(ii) antibody exhibited higher sensitivity (i.e., steeper slope) to the A/Indonesia/05/2005 antigen than the H5(i) and H5(iii) capture mAbs (Fig. 4a). Similarly, the H7(ii) antibody exhibited higher sensitivity to the A/Shanghai/02/2013 antigen than the H7(i) antibody (Fig. 4c). This is not unexpected as different antibodies often have very different binding constants. Because the assay utilizes a calibration curve, the differences in antibody binding constants does not result in different concentrations determined by each antibody. Using each calibration curve, LDRs were calculated and reported in Table 1. The LDRs are ≥150-fold for all capture antibodies and are at least 15 times greater than the quantification range of SRID (~6–30 µg/mL).

**Table 1:** Quantification ranges for VXI-pHA and SRID

Subtype	mAb ID	Lower QL (µg/mL)	Upper QL (µg/mL)	Range
H5	H5(i)	0.003	1	300×
	H5(ii)	0.001	0.5	500×
	H5(iii)	0.002	1	500×
	H5(iv)	0.001	0.5	500×
	H5(v)	0.001	0.5	500×
	SRID	5.3	28.7	5.4×
H7	H7(i)	0.002	0.75	375×
	H7(ii)	0.001	0.75	750×
	SRID	5.4	31.3	5.8×
H9	H9(i)	0.005	0.75	150×
	H9(ii)	0.004	0.75	187×
	SRID	5.3	>40.6	>7.7×

### VXI-pHA is a more sensitive potency assay than SRID

Three of the four antigens (A/H5 A/Indonesia, A/H7 A/Shanghai, and A/H9 A/chicken/HK) used to assess the LDR of VXI-pHA were analyzed by SRID with appropriately matched reference antisera to provide a side-by-side comparison of the sensitivity of the two assays. The A/H5 A/gyrfalcon sample was not analyzed by SRID because at this time there are no SRID reference reagents available. Eight antigen concentrations were tested, from 0.5 to 40 µg/mL of HA, to test the lower and upper limits of the SRID assay. A representative SRID response curve for the H7 antigen is presented in Fig. 4e, f. For each antigen tested, the LDR of the assay was determined using a previously reported method.^9^ Briefly, a linear regression was fit to the points corresponding to the reported LDR for SRID^5^ (red points in Fig. 4e). Next, the measured diameter for each concentration tested was compared to the expected diameter based on the linear regression using a one sided, one-sample *t*-test (*p* < 0.05) to determine if any given data point significantly deviated from the regression. Concentrations that significantly deviated from the regression were considered outside the LDR of the assay. Using this method, the LDRs for H5, H7, and H9 SRID assays were calculated and are presented in Table 1. For H5 and H7, the reported LDR of the SRID assay remained 5–30 µg/mL and was not expanded. For H9, the 40 µg/mL concentration did not significantly deviate from the linear regression, and thus the upper limit of quantification for H9 was determined to be ≥40 µg/mL. Concentrations higher than 28 µg/mL of reference antigen A/H5 A/Indonesia were not tested due to a low initial antigen concentration (32 µg/mL).

The LDR for each of the VaxArray capture antibodies was compared to the measured LDRs for SRID (Table 1). For H5, the SRID LDR was 5.4× whereas the VaxArray LDRs were 300×, 500×, and 500× for the H5(i), H5(ii), and H5 (iii) antibodies, respectively. For H7, the SRID LDR was 5.8× whereas the VaxArray LDRs were 375× and 750× for H7(i) and H7(ii) antibodies, respectively. For H9, we could not calculate an SRID LDR because we did not define an upper limit of the assay. In further comparing the two assays, the average lower quantification limit for VaxArray was 2650-fold lower than for SRID (0.002 ± 0.001 µg/mL compared to 5.3 ± 0.1 µg/mL for SRID). The lower limits of quantification greatly reduce the amount of sample required compared to SRID, a crucial benefit for quantification of dose-sparing vaccines and/or vaccines in high demand. Additionally, the large dilution factors allowed by this low limit of quantification can greatly dilute out interfering substances such as crude matrix proteins and adjuvants, all known inhibitors of the SRID assay.^5^

### VXI-pHA demonstrates a high level of accuracy

To assess the accuracy of the VXI-pHA assay, four different production lots of an A/Hong Kong/125/2017 H7N9 monobulk drug substance were evaluated by VXI-pHA following the standard procedure. At the time of this study, SRID reference reagents were not available for this new “5^th^ wave” H7 influenza vaccine in development and could not be used as a reference standard. To overcome this hurdle, the purity-adjusted bicinchoninic acid (paBCA) assay was used to determine total HA protein content of one of the production lots. This sample was determined to have 154 ± 17 µg/mL HA and was then used as an internal standard to calibrate the VXI-pHA assay. This approach is not unreasonable, since a purity-adjusted total protein measurement is used to define the HA concentration within the Primary Liquid Standard used by WHO laboratories to calibrate reference reagents.^10^

**Fig. 5:**
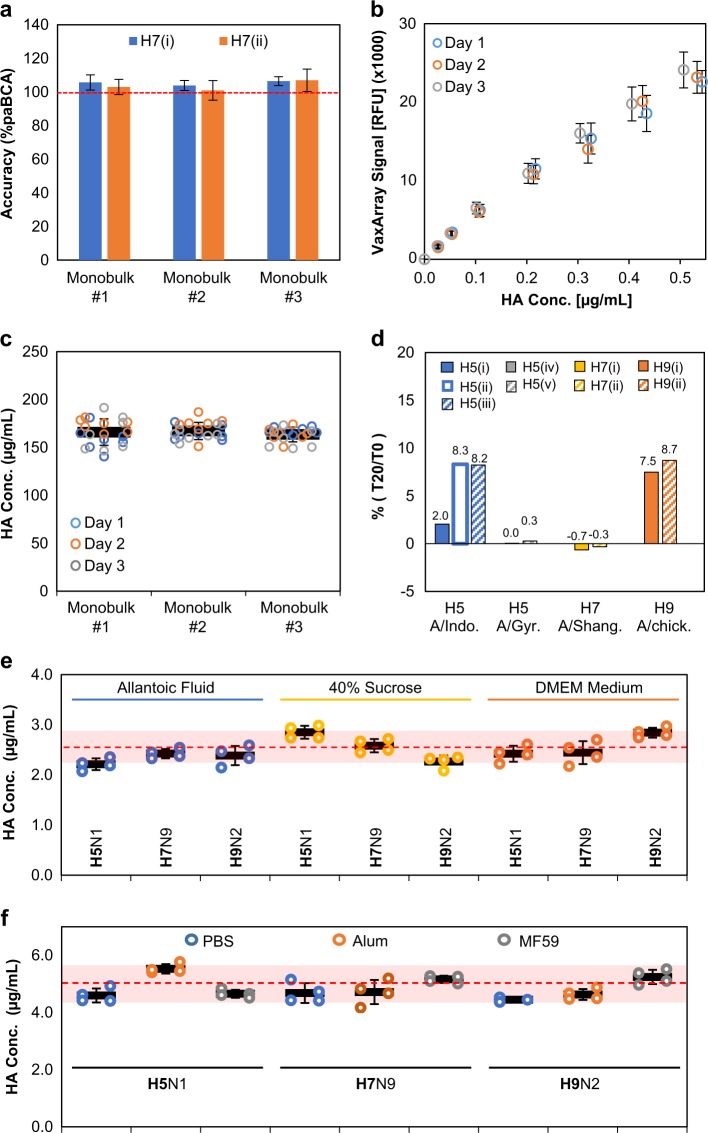
Analytical performance of VXI-pHA. **a** Accuracy. Average of the VXI-pHA potency measurements divided by the average of the paBCA measurements (i.e., % of paBCA) for three H7N9 monobulk samples from three different production runs utilizing the H7(i) capture antibody (blue, filled bars) and the H7(ii) capture antibody (orange, filled bars). The VXI-pHA assay was run on three separate days in replicate of 7. The paBCA assay was run on three separate days in replicate of 3. Error bars represent the relative standard deviation of the VXI-pHA measurements. **b** Precision. Calibration curves generated from an H7N9 internal standard on three different days utilizing the H7(i) capture antibody. Data from each day are represented as a different color. Error bars represent the standard deviation of the 9 antibody spots. **c** Precision. Univariate scatterplot representation of VXI-pHA potency values for three different H7N9 monobulk samples from three different production runs. Each data point is colored in accordance with the day it was analyzed. Thick black bars represent average HA concentration for each monobulk sample across all 3 days. Error bars represent error across all 3 days for each monobulk sample. **d** Stability indication capabilities of VXI-pHA assay. Samples were analyzed via VXI-pHA in triplicate before (*T*0) and after a 20 h incubation at 56 °C (*T*20) and %*T*0 values were calculated. Results are presented as %*T*0 (*T*20/*T*0) for each capture antibody (see legend). Antigens used are shown on the *x*-axis. **e** VXI-pHA potency determination of solutions containing HA antigens spiked into allantoic fluid, 40% sucrose, and used DMEM medium from uninfected MDCK cells. The following antigens were used: A/Indonesia/05/2005 CBER Lot #83 (H5N1), A/Shanghai/02/2013 CBER Lot #78 (H7N9), and A/chicken/Hong Kong/G9/1997 NIBSC Lot 08/228 (H9N2). **f** VXI-pHA potency determination for antigens spiked into adjuvants. Antigens were mixed with 1.7 mg/mL elemental aluminum or 19.5 mg/mL MF59 for a final HA concentration of 2.5 µg/mL before being diluted and analyzed by VXI-pHA. For each sample, a PBS negative control was included. Antigens included A/Indonesia/05/2005 CBER Lot #83 (H5N1), A/Shanghai/02/2013 vaccine (H7N9), and A/chicken/Hong Kong/G9/1997 Lot # U51P72H1 from BEI (H9N2). For both **e** and **f**, each data point represents a single replicate. The thick black bar represents the average across the four replicates. Error bars represent the standard deviation across the four replicates for each sample. The red-dotted line represents the expected concentration for each sample. The shaded red region represents the expected concentration plus and minus the error associated with the assay

Using the internal standard for calibration, three other production lots of the monobulk drug substance were analyzed by the VXI-pHA assay using both H7 antibodies (i and ii) in replicates of seven on three separate days. The same three production lots were also analyzed on three separate days in replicates of three for HA content by the paBCA method. The accuracy of the VXI-pHA measurements was assessed by comparing the concentrations determined by VXI-pHA to those determined by paBCA and was defined as “percent of paBCA”. By comparing VXI-pHA to paBCA, the assumption must be made that the protein content of the monobulk is 100% intact as the paBCA method is a total protein measurement and not able to distinguish between intact and degraded protein. The percent agreement between methods for the three monobulk production lots is 105%, 104%, and 106% for the H7(i) VXI-pHA capture antibody and 103%, 101%, and 107% for the H7(ii) VXI-pHA capture antibody (Fig. 5a). Note the H7(ii) VXI-pHA capture antibody performed very similarly and within error of H7(i) demonstrating that either antibody can be used to quantify H7 antigen.

### VXI-pHA demonstrates a high level of precision

The data generated in the accuracy study also included an assessment of precision since three production lots of the monobulk drug substance were analyzed by VXI-pHA in replicates of seven on three separate days. In each analysis, an 8-point calibration curve was run. The calibration curves generated on each day were compared (Fig. 5b) and the slope and associated error were found to be within error of one another demonstrating the high reproducibility of the assay. The replicate analysis resulted in the following HA concentrations of the three monobulk formulations: 185 ± 15, 186 ± 10, 182 ± 9 µg/mL (Fig. 5c). The intraday assay precision was 5%, 5%, and 7% for day one, two, and three, respectively (Table 2). The average error across all replicates tested was 6% for the three samples. For comparison, the average relative error reported for the SRID assay is 12%;^5^ thus, VXI-pHA offers improved precision for the quantification of potency.

**Table 2:** Summary of intraday precision, interday precision, and pooled replicate variation

	Intraday replicate variation (%CV)			Interday average variation (%CV)	Pooled replicate variation (%CV)
Sample:	Day 1	Day 2	Day 3	*–*	*–*
	*n* *=* *7*	*n* *=* *7*	*n* *=* *7*	*n* *=* *3*	*n* *=* *21*
Monobulk #1	8	4	11	4	8
Monobulk #2	4	6	4	3	5
Monobulk #3	2	5	6	3	5
Average %CV	5 ± 3	5 ± 1	7 ± 3	3 ± 1	6 ± 2

### VXI-pHA is capable of detecting changes in protein conformation

Vaccine manufacturers are required to monitor the potency of their vaccines at batch release and over time after release. Additionally, due to the need to release pandemic vaccines as quickly as possible, manufacturers often perform forced degradation studies to predict vaccine stability before batch release. To test the stability indication properties of the VXI-pHA assay, we performed a representative forced degradation experiment for a panel of antigens designed to address the stability indication capabilities of each capture antibody. Briefly, samples were analyzed in triplicate by VXI-pHA and SRID before (*T*0) and after 20 h at 56 °C (*T*20) and %*T*0 values were calculated (*T*20/*T*0, represented as a percentage).

After a 20 h incubation at 56 °C the VXI-measured HA potency for each sample decreased to less than 9% of the non-degraded HA concentration. Specifically, the %(*T*20/*T*0) was <8.3% for H5 A/Indonesia/05/2005, <0.3% for H5 A/gyrfalcon/Washington/41088-6/2014, ~0% for H7 A/Shanghai/02/2013, and <8.7% for H9 A/chicken/Hong Kong/G9/1997 (Fig. 5d). The differences between H5(i) and H5(ii and iii) are likely due to differences in the stability of the different epitopes that the antibodies probe. The epitope detected by H5(i) is likely much more sensitive to heat than the epitopes probed by the other two antibodies. The same antigens were force degraded and analyzed by SRID. After 20 h at 56 °C, none of the antigens produced signals above the lower limit of quantification for SRID. Therefore, both potency assays demonstrated the ability to detect changes in protein conformation upon forced degradation. While real-time studies under a variety of conditions are needed to fully quantify the stability indication capabilities of the VXI-pHA assay, the studies presented here demonstrate the utility of the VXI-pHA assay in monitoring stability in forced degradation studies similar to those performed by vaccine manufacturers.

### VXI-pHA is compatible with in-process production samples

We previously demonstrated that the VaxArray Influenza Seasonal Hemagglutinin Potency Assay could quantify both recombinant and egg-derived HA in crude extracts such as cell-culture media and allantoic fluid.^6,7^ To evaluate performance of the VXI-pHA for crude samples, we spiked reference reagents for A/Indonesia/05/2005 (H5N1, CBER Lot #83), A/Shanghai/02/2013 (H7N9, CBER Lot #78), and A/chicken/Hong Kong/G9/1997 (H9N2, NIBSC Lot #08/228) into crude matrices to simulate in-process sample types throughout manufacturing. The crude matrices tested included (i) allantoic fluid to mimic conditions at harvest from eggs, (ii) exhausted tissue culture medium to mimic conditions at harvest from tissue culture, and (iii) 40% sucrose to mimic the product isolated during sucrose gradient purification. Antigens were spiked into each matrix at a final concentration of 2.5 µg/mL HA.

The average HA concentration determined by VXI-pHA for each simulated in-process sample was compared to the expected sample concentration of 2.5 µg/mL. In Fig. 5e, the calculated HA concentration is plotted for each replicate. A tight clustering of replicates near the expected concentration of 2.5 µg/mL was observed and the averages for each sample were within 10% of the expected concentration (red shading in Fig. 5e). These studies suggest that the crude matrices found in in-process samples do not interfere with the assay and that VXI-pHA is capable of accurately measuring HA potency (within 10%) for in-process samples. In comparison, the SRID assay is not capable of reliably monitoring HA potency at low concentrations in crude in-process samples.^5^ For this reason, techniques like high-performance liquid chromatography (HPLC) are often used to monitor yield at the early stages of vaccine production. HPLC, however, does not measure active, potent HA, but rather total HA. VXI-pHA represents a high-throughput alternative measurement that can be performed within 2 h to allow vaccine manufacturers to track the yield of conformationally active, immunogenic HA from the early to the late stages of pandemic vaccine production pipeline.

### VXI-pHA is compatible with dose-sparing adjuvanted vaccines

To evaluate the utility of the VXI-pHA assay for quantifying the potency of dose-sparing, adjuvanted vaccines, we formulated mock dose-sparing, adjuvanted vaccines containing industry-relevant adjuvants and analyzed them via VXI-pHA. Each mock vaccine consisted of a final concentration of 5 µg/mL of HA and 1.7 mg/mL elemental aluminum or 19.5 mg/mL squalene (from MF59). A non-adjuvanted negative control sample was included where phosphate-buffered saline (PBS) was added instead of adjuvant. Monovalent mock-formulated dose-sparing vaccines were generated for the following strains: A/Indonesia/05/2005 (H5N1), A/Shanghai/02/2013 (H7N9), and A/chicken/Hong Kong/G9/1997 (H9N2). Each monovalent mock-formulated vaccine and negative PBS control was properly diluted to within the LDR of the VXI-pHA assay and analyzed against a corresponding, non-adjuvanted 8-point calibration curve. The expected concentration for all samples was 5.0 µg/mL (demonstrated by the red-dotted line in Fig. 5f). All samples, despite subtype and adjuvant-type, were measured on average to be within 10% of the expected concentration (red shading in Fig. 5f). The differences between samples is likely due to some noise introduced during sample preparation (i.e., pipetting) and is not due to the presence of adjuvant as the noise is also evident in the PBS, non-adjuvanted sample. These finding suggest that the VXI-pHA assay is a reliable measure of potency (±10%) even in the presence of aluminum hydroxide- or squalene-based adjuvants.

## Discussion

The development of vaccines against potentially pandemic influenza strains is a critical piece in the global response to an influenza pandemic. Immediate access to a potency assay for monitoring of immunogenic HA concentrations would streamline the development, production, release, and ongoing stability monitoring of pandemic vaccines, such as those stored in the National Influenza Vaccine Stockpile.^11^ To address the current bottleneck in influenza vaccine potency determination, an on-demand potency assay was developed for influenza vaccines produced in response to the most concerning influenza A subtypes. Specifically, new strains of avian influenza H5 and H7 subtypes continue to infect humans who work closely with poultry, which has led the WHO to recommend the development of new CVVs to address divergence from older CVVs.^8^

The VXI-pHA assay relies on a panel of monoclonal antibodies designed to cumulatively detect HA from H5, H7, and H9 strains spanning several years despite antigenic drift in the influenza HA protein. Overall, the panel of anti-H5, H7, and H9 mAbs selected for inclusion in the VXI-pHA assay exhibits good specificity, high sensitivity, and broad coverage over a large span of viral evolutionary time including nearly all the proposed CVVs. Interestingly, HA from recently isolated A/H7N9, which resulted in the recently announced A/Guangdong and A/Hong Kong CVVs,^8^ are detected by the same antibodies that detect the much more distant A/H7N7 and A/H7N2 strains of influenza, indicating binding to a relatively conserved epitope. The robust responses for each subtype offers a measure of confidence that the test would be viable over some degree of HA protein evolution.

While the VXI-pHA assay has been developed to be fairly resistant to evolutionary change by probing more than one relatively conserved epitope for each subtype, it is possible that a new strain could arise that the VXI-pHA assay is not able to detect. In the event that a new strain is not detected on the current version of the array, InDevR has developed a protocol to rapidly screen all available mAbs in a highly multiplexed format. If an mAb exists that can detect the new strain, it can be incorporated into the array, verified, and validated in an expedient, but quality managed, process. If no mAb exists, InDevR would leverage close collaboration with a number of government agencies to develop an appropriate mAb on an accelerated time scale. As a fall-back position for emergency use, we are also investigating the possibility of incorporating “universal” mAbs for detection and quantification.^12^ While these mAbs do not offer subtype specificity, that level of specificity may not be needed for a monovalent pandemic vaccine.

The VXI-pHA assay also has broad coverage across different vaccine production methods. Historically, influenza vaccines have been mass produced in embryonated chicken eggs, but recent developments in cell-culture based and recombinant protein production platforms have broadened the potential sources of vaccine antigens. Data reported here demonstrate that the VXI-pHA assay is capable of being used to determine the potency of novel, recombinant, and cell-based vaccines in addition to traditional, egg-based vaccines, thus overcoming the incompatibility issues that manufacturers of novel vaccines such as rHA vaccines and virus-like particle (VLP) vaccines have faced with SRID.^13^

Many of the benefits of the VXI-pHA assay are due to the broad LDR of the assay which is at least 15 times greater than the quantification range in SRID (~6–30 µg/mL). Additionally, the lower limit of quantification for the assay greatly reduces the amount of sample required compared to SRID, a crucial benefit for quantification of dose-sparing vaccines and/or vaccines in high demand. Additionally, the large dilution factors allowed by this low limit of quantification can greatly dilute out interfering substances such as crude matrix proteins and adjuvants, all known inhibitors of the SRID assay.^5^ Common adjuvants such as aluminum hydroxide and squalene-based adjuvants like MF59 are used to deliver antigen dose-sparing vaccines while maintaining the efficacy of larger doses, thereby enabling broader protection of the population. Due to the inherent incompatibility of SRID with some adjuvants, as well as concern over the stability of antigen in the presence of adjuvants over time, most adjuvanted vaccines are currently stored separately from adjuvants in two vials and mixed bed-side before administration to patients. Because the VXI-pHA can be applied to low-dose adjuvanted vaccines, it could be utilized to monitor potency in the presence of adjuvants. With more information at hand on the stability and potency of vaccines stored in adjuvants for extended periods of time, the need for the two-vial system could be reassessed.

The major time benefit of VXI-pHA over SRID is that the assay is not reliant on the time-consuming production process for generating reference reagents for calibration of the assay. Our approach to calibrating the VXI-pHA assay in this study was to utilize an internal standard by quantifying total HA using the paBCA method. In the case of a pandemic, it is likely that vaccine manufacturers would take a similar approach for tracking HA concentration during vaccine development.

There is increasing scientific evidence that neuraminidase (NA) within influenza vaccines leads to NA immunity, decreased viral shedding, and reduced severity of influenza disease.^14–17^ For this reasons, we have developed a VaxArray assay for the quantification of NA in seasonal influenza vaccines. The assay utilizes the same technology as VXI-pHA, which is made modular by simply changing the capture and label antibodies for NA-specific reagents. Future efforts will expand the technology to include an assay for pandemic NA, allowing manufacturers to track both HA and NA in pandemic vaccines in rapid fashion.

In summary, the VXI-pHA assay is reagent sparing, stability indicating, and capable of accurately and precisely monitoring vaccine potency of low-dose, adjuvanted pandemic vaccines. The VXI-pHA assay is also capable of monitoring HA concentration in crude in-process samples, potentially allowing manufacturers to increase vaccine yield, and ultimately the amount of life-saving vaccines they are able to produce.

## Methods

### VXI-pHA standard procedure

The VXI-pHA technology is similar to the VaxArray Influenza Seasonal Hemagglutinin Potency Assay (VXI-sHA) described previously.^6,7^ VXI-pHA reagent kits (Cat# VXI-7200; InDevR Inc.) contain two microarray slides, printed with 16 replicate arrays per slide, Fiducial Detection Label, Protein Blocking Buffer (PBB), and two Wash Buffers. Prior to use, VXI-pHA slides were removed from the refrigerator and equilibrated to room temperature for 30 min in the provided foil pouch. Samples were prepared individually by lysing at room temperature for 30 min in the presence of 1% Zwittergent 3–14 (Cat# 693017; EMD Millipore). Each sample was further diluted in Protein Blocking Buffer plus 1% Zwittergent 3–14 (PBB/Z) and 50 µL was applied to individual arrays on the slide following the method described in the VaxArray Influenza Potency Assay Operation Manual (Rev. 001). After a 1 h incubation at room temperature in a humidity chamber (Cat# VX-6200; InDevR Inc.), sample was removed using an 8-channel pipette. A label mixture of PBB, Fiducial Detection Label, and antigen detection label was prepared and aliquoted into 8-tube PCR strips. After removal of antigen from the slide, 50 µL of label mixture was added to each array using an 8-channel pipette. Each slide was further incubated in the humidity chamber for 30 min before subsequent, sequential washing in Wash Buffer 1, Wash Buffer 2, 70% Ethanol, and ultrapure water. Slides were dried using a compressed air pump system, imaged using the VaxArray Imaging System (Cat# VX-6000, InDevR Inc.), and raw fluorescent signal intensity of the printed capture antibody spots extracted. Data were automatically processed using either an excel spreadsheet (slightly modified version of the VaxArray Processing Workbook v1.2 described by Kuck et al.^7^) or the VaxArray Analysis Software Package which utilizes the same algorithm described by Kuck et al.^7^ For each capture antibody, calibration curves were plotted using antigen concentration and median signal intensity of the nine antibody replicate spots on each individual array, and, when appropriate, the HA concentration of samples was automatically calculated against the standard.

### Strain coverage and specificity

Reactivity and specificity for each of the mAbs included on the array was evaluated against the panel of H5, H7, and H9 antigens listed in Fig. 2. Most of the antigens were acquired from publicly available sources such as the BEI, IRR, CBER, and the National Institute for Biological Standards and Control (NIBSC). The remaining antigens were provided by our collaborators who granted permission to share these results. All antigens were diluted to 0.5 µg/mL HA protein, except for recombinant proteins which were diluted to 5 µg/mL, and analyzed by VXI-pHA using the standard procedure described above. A “universal” polyclonal label antibody (Polyclonal A/B Label, Cat# VXI-7601, InDevR) was used to quantify all egg-based HA samples. A broadly reactive monoclonal label more compatible with recombinant and cell-based antigens (Cat# VXI-7604, InDevR Inc.) was used to detect H5 and H9 recombinant and cell-based samples, and a separate monoclonal label (Cat# VXI-7605, InDevR Inc.) was used to detect H7 recombinant and cell-based samples. The need for different labels is possibly due to differences in glycosylation patterns. The “universal” polyclonal antibody was raised against egg-derived antigens and has consistently under performed in labeling cell-derived antigens. The monoclonal capture and label antibodies were previously developed for broad detection regardless of antigen production method.

For each antigen tested, the VaxArray Analysis Software output raw fluorescence intensities for each capture antibody and for the array background. An average background intensity was calculated for all arrays used in the experiment. Using this value, 3×, 10×, 20×, and 40× background cut offs were calculated and relative intensities for each capture antibody against each antigen was characterized.

### VXI-pHA quantification range

HA-containing antigens appropriate for evaluating the quantification for the panel of nine capture mAbs (H5 A/Indonesia (CBER, Lot #50), H5 A/gyrfalcon (Cat# FR-1418, IRR), H7 A/Shanghai (CBER, Lot #78), and H9 A/chicken/Hong Kong (NIBSC, Lot #08/228)) were lysed for 30 min in PBS plus 1% Zwittergent 3–14. Each antigen was serially diluted monovalently in PBB/Z over a range of 13 concentrations and analyzed in the VXI-pHA assay with 3 additional PBB/Z-only arrays (antigen-blanks). The VaxArray Analysis Software output raw fluorescence intensities for each capture antibody and for the background on each array, including the antigen-blank arrays. The median VaxArray signal for each antibody in the array was plotted against the HA concentration. The HA quantification range for each capture antibody was determined by calculating linear ranges in four adjacent-point intervals across the 13 point standard curve (10 antigen-only ranges plus 3 antigen blank containing ranges). The upper limit of the quantification was defined as the highest concentration in the final range with an acceptable linear fit (*R*^2^ of >0.95). The lower limit of quantification for each antibody, defined by the median signal of the capture antibody spots in the antigen blank plus five times the standard deviation across these spots, was calculated individually for each capture antibody with each antigen blank and then averaged.

### Stability indication studies

Antigen samples were diluted in PBS to a final concentration of 15 µg/mL HA and 100 µL aliquots were added to 1.5 mL amber glass vials, sealed with a crimp top, and weighed. Samples were heated in a water bath for 20 h (*T*20) while a control was retained at 4 °C (*T*0). The water temperature was continuously monitored and was 55–56 °C during the entire degradation time period. After degradation, the vials were briefly cooled on ice and then stored at 4 °C until analysis later that day. Each vial was re-weighed before analysis to check for possible evaporation during degradation. All weights showed <0.07% difference after degradation. Samples before (*T*0) and after 20 h at 56 °C (*T*20) were analyzed in triplicate with an 8-point standard curve of non-degraded antigen using the VXI-pHA potency assay using the standard procedure described above.

### Quantification of HA in crude matrix

HA-containing antigens were spiked into a 40% sucrose solution (Cat# S9378; SigmaAldrich), allantoic fluid from 10-day-old embryonated chicken eggs (Cat# BV027; Virapur), and exhausted DMEM + 10% fetal bovine serum tissue culture media taken from non-infected MDCK cells (provided by a collaborator) to mock 2.5 µg/mL HA solutions in each matrix. All spiked samples were lysed with 1% Zwittergent 3–14 for 30 min and diluted in PBB/Z to a final expected concentration of 0.1 µg/mL HA and analyzed by VXI-pHA in quadruplicate (*n* = 4) using the standard procedure described above. A standard curve of antigen was prepared by lysing the same antigens in PBS and 1% Zwittergent, serially diluting in PBB/Z, and was analyzed alongside the crude-matrix spike antigens by VXI-pHA. The HA concentration in each spiked sample was determined against the appropriate calibration curve. The average HA concentration determined for each spiked sample was compared to the expected sample concentration based on the known concentration of the antigen and the performed dilutions. The percent difference between the expected and measured HA concentrations was calculated for each sample.

### Quantification of HA in mock dose-sparing, adjuvanted vaccines

Three HA-containing antigens were individually spiked into aluminum hydroxide (Cat# 77161; ThermoFisher), MF59 (Cat# Vac-adx-10; Invivogen), and PBS at final concentrations of 5.0 µg/mL of HA resulting in two “mock” adjuvanted vaccines with adjuvant concentrations of 0.17 mg/mL of elemental aluminum or 1.95 mg/mL squalene and a negative PBS control. Each solution was incubated at room temperature for 30 min in adjuvant or PBS before being lysed with 1% Zwittergent for 30 min. The “mock” adjuvanted vaccines and the corresponding negative controls were diluted in PBB/Z to 0.1 µg/mL for the H5 A/Indonesia sample (CBER, Lot #83) and 0.25 µg/mL HA for the H7 A/Shanghai (CBER, Lot #78) and H9 A/chicken/Hong Kong (Cat# NR-12140, BEI) sample and analyzed by VXI-pHA in quadruplicate (*n* = 4) against an eight-point calibration curve of a matched non-adjuvanted antigen. Using the standard VaxArray procedure, average HA concentrations were determined for each “mock” adjuvanted vaccine and negative control using the appropriate standard curve. The HA concentrations of each sample were compared to the expected HA concentration, based upon the known dilution factors, to investigate the effect of aluminum hydroxide and MF59 on VXI-pHA performance compared to the negative control.

### Single radial immunodiffusion

SRID was performed as described previously^18^ with minor deviations. Three separate agarose preparations were prepared by dissolving 1.1 g of agarose (Cat# 50011; Lonza) in 100 mL of PBS and kept at 55 °C. Antisera solutions for H5 A/Indonesia (CBER, Lot# H5-Ab-1217), H7 A/Shanghai (CBER, Lot# H7-Ab-1402), and H9 A/chicken/Hong Kong (NIBSC, Lot# 08/202) were reconstituted in ultrapure water according to their associated instructions and added to the agarose solutions. For each subtype, the agarose solution was swirled to mix and 25 mL was added to gelbond film (Cat# 53734; Lonza) and allowed to cool. After solidifying, 25 4-mm holes were punched into each gel using a biopsy punch (Cat# 96–1115; Sklar Instruments). Each sample to be evaluated was lysed in PBS and 1% Zwittergent 3–14 at room temperature for 30 min. Further dilution in PBS plus 1% Zwittergent (PBS/Z) was used to generate each standard curve. To each well of the appropriately matched gel 20 µL of sample was added. Three replicates of each sample were analyzed by each gel along with one PBS/Z antigen blank. Gels were incubated in a covered humidity chamber for 16–18 h (overnight) at room temperature. After incubation, each gel was washed with 0.9% sodium chloride solution (Cat# 0241, Amresco), rinsed with ultrapure water three times, pressed, and dried in an incubator set to 55 °C. Each gel was stained by Coomassie Brilliant Blue R250 (Cat# B0149; SigmaAldrich) followed by destain in a 30% methanol (Cat# 3016; Mallinckrodt) and 12% acetic acid (Cat# V193; Mallinckrodt) solution until background color had sufficiently reduced. Stained gels were dried in a 55 °C incubator and then scanned using an HP benchtop scanner. The circumference of each spot was measured using ImageJ and converted into the associated spot diameter. Spot diameters were plotted against the corresponding HA concentration. A linear fit was applied to the standard quantification range of 5–30 µg/mL HA spots and the linear slope and intercept calculated.

### Purity-adjusted total protein by bicinchoninic acid assay

For each H7N9 monovalent drug substance, the total protein content was determined by the microbicinchoninic acid (BCA) assay (Cat# 23235; ThermoFisher). Next, 20 µL of each sample were denatured and reduced at 95 °C for 5 min followed by deglycosylation in the presence of PNGase F (Cat# V4831; Promega) overnight at 37 °C. The XCell *SureLock* gel box and NuPAGE™ pre-cast 4–12% bis-tris gradient gels (Cat# NP0322; ThermoFisher) were used to evaluate each deglycosylated monobulk sample alongside a non-treated monobulk to evaluate band shifts. Gels were stained with Coomassie® and imaged with an Olympus camera. Protein content of HA bands was calculated by applying the relative densitometric peak area of both HA1 and HA2 bands in the deglycosylated lanes to the total protein content determined by the BCA assay.

### Code availability

The mathematically algorithm used to analyze the VXI-pHA assay can be accessed in a previously published study.^7^

## Data Availability

All relevant data from this study are available from the authors.
